# Hospital and Institutional News

**Published:** 1913-03-22

**Authors:** 


### HOSPITAL AND INSTITUTIONAL NEWS.

### THE WORD "CHARITY" ON WORKMEN'S TICKETS

Misconception still continues to exist, especially in the Midlands and the North, as to the relation of working-men's subscriptions to hospital treatment. For example, only last week a discussion took place at a governors' meeting of the Hartlepools Hospital over the word "Charity" on workmen's tickets. Mr. Charles Sargeant, on behalf of a local firm's employees, moved that the word be deleted from the tickets issued to working-men subscribers. Alderman Hunter, however, pointed out that the workmen had a wrong conception of the word altogether. The workmen's subscriptions did not account for all the money spent on the hospital. The matter was relegated to the house committee. This question has caused heart-searchings before now, but Alderman Hunter was certainly on the right track. The working men—whose subscriptions, as they surely must realise, go to aid an institution which treats not only workmen but many others who are sick—are giving as well as taking charity: but in so far as the institution is not exclusively for them, and as their subscriptions do not represent the whole cost of their treatment in the institution, the word "Charity" should be retained, for it marks their contribution to the needs of others, and points to an institution which does not pretend to exact the cost of treatment from the ordinary patient admitted to its wards.

### THROAT HOSPITALS AND THE CLERICAL VOICE.

A new *rôle* for throat and ear hospitals was suggested last week by Dr. Russell Wakefield, Bishop of Birmingham, at the annual meeting of the Midland Ear and Throat Hospital in that city. The Bishop remarked that voice-production was extremely carelessly considered, and that the throats, and he might have added the audiences, of many people who spoke in public suffered in consequence very greatly indeed. In Germany and elsewhere abroad certain religious and other bodies had a system of teaching voice-production which was very useful in the care of the throat. "I am not at all sure," added the Bishop, "that it would not be a good thing, if the finances improved, for classes to be held in connection with the hospital to teach people how to speak." No doubt, what has recently been termed in the lay press the clerical voice was in the Bishop's mind as he made this suggestion. On his proposal two things may be said. The first concerns that particular assumed droning which is what everyone knows to be meant by the clerical voice. We do not think sufficient attention has been paid to its true cause, which is not primarily want of education, absence of training, or personal vanity on the part of most preachers, but simply the fact that a large part of their public speaking consists in the constant repetition of certain well-known literary formulæ, as the collects and prayers may be rightly termed in this connection from a medical point of view. The result is that the majority of men almost inevitably are compelled to react against the verbal monotony by a sub-conscious attempt to vary it in the only way possible—that is to say by strange inflections of the voice, or in extreme cases by actual mispronunciation. Thus we get acknōwledge for acknowledge—so much ridiculed in the Concise Oxford Dictionary. Only a few men are invariably open to the beauties of well-known passages of literature; when they cease to be alive to them monotony ensues, and affectations of voice are the inevitable reaction. As to Dr. Wakefield's suggestion that throat hospitals should hold classes for voice-production, we doubt on many grounds if this is practicable, though we invite expressions of opinion from the administrative and medical staffs of the institutions concerned. We incline to think that such classes could more conveniently be held in connection with school clinics or similar bodies, which Birmingham has set such a magnificent example in linking up into one municipal system.

### SECRET IMPORTATION OF MENTAL PATIENTS.

At a committee meeting of the Clonmel Mental Hospital, Ireland, last week the resident medical officer, Dr. Harvey, drew attention to the increasing numbers of mentally afflicted persons who are being deported from the United States to Ireland. Since the last meeting, he stated, two such persons had arrived at the mental hospital—a man and a girl—under circumstances which called for immediate consideration. The man had actually been for fifty years in America and had been put, apparently by agents employed for the purpose, into the train at Queenstown and then left to find his own way back to the institution. The case of the girl, also insane, was still more remarkable. She had been sent home from America without notice, and was found by her parents in their yard one morning. Here, again, it would seem that agents accompanied the patients to Ireland and then turned them loose to find their way to the institution or their own home. Dr. Harvey has done quite right to expose this extraordinary system, which has nothing to recommend it but the callousness of those who seek thus to rid themselves of human wreckage. It is urgently necessary that the whole system of secret agents and its authors should be exposed, so that those responsible for it can be brought to book without delay. We trust that Dr. Harvey will not let matters rest and invite him to co-ordinate his experiences in our columns, so that steps may be taken at once to regulate this secret system of deportation to Ireland.

### A VICAR FORGES A MATRON'S SIGNATURE.

After a protracted trial judgment has been given against the Rev. James Wilson Alexander Mackenzie, Vicar of Whitwick, Leicestershire, on the charge of issuing a forged promissory note for £300. It will be remembered that the charge preferred against Mr. Mackenzie has a special interest for institutional workers from the fact that the note purported to bear the signature of the late Miss Murray, matron of Ramsey Isolation Hospital. The accused refused to go into the witness box, and Deemster Moore remarked in his summing up that from this the jury could draw no conclusions. They found Mr. Mackenzie guilty, however, and he was sentenced to twelve months' hard labour. A feature that hardly requires emphasising is the rareness with which institutional administrators are connected, however indirectly, as in this case, with trials of this kind.

### A NEW MISUSE FOR MEDICAL CERTIFICATES.

This rather unusual case, *Latham* v. *Stevens*, which occupied the attention of the Courts last week, raised an issue with regard to medical certificates which we do not remember to have heard debated before. The defendant went to the plaintiff and told him that he had at some previous date been suffering from pulmonary tuberculosis; that, although quite recovered, some of his friends and associates regarded him with aversion as a possible source of tuberculous contagion; and that he wanted a certificate to the effect that he was no longer capable of infecting his fellow-creatures. On these representations the plaintiff examined the defendant, and found no signs of active tuberculosis, nor did the sputum contain any bacilli. Accordingly he drew up a certificate on lines suggested by the defendant. It turned out that in reality the defendant required this certificate for the purpose of advertising a secret consumption cure, and not at all for the purposes he mentioned to the plaintiff. The latter accordingly objected to the use of his name and certificate by the defendant and his limited company; but the latter contended that, as they had bought and paid for the certificate, they had a right to use it as they thought fit. The upshot of the subsequent litigation was to uphold the plaintiff's objection. But the case clearly shows what great care is necessary in drawing up and signing any document which may be used in ways undreamed of by the writer. In this case the plaintiff's object was attained; but it is easy to see that a very little carelessness in signing such a certificate might result in very mortifying results to the certifier.

### CARDIFF HOSPITAL AND PANEL APPOINTMENTS.

At the monthly meeting of the Board of Management of King Edward VII.'s Hospital, Cardiff, it was unanimously decided to interpret the rule, passed in 1908, prohibiting members of the honorary medical staff from holding club appointments, as having reference also to panel appointments.

### HINTS TO TESTATORS.

If one thing more than another has been dinned into hospital managers, it is that the testator, as a class, is a person whose benevolence is rarely equalled by forethought in the necessary means for carrying it out. A bequest to a hospital whose title in the deed of gift was expressed with such remarkable ambiguity as to bring half a dozen claimants into the Courts is still in recent memory, and this is often a type of the difficulties which testators are not scrupulous to avoid. It may seem then a bold task to present intending testators with a somewhat nice intellectual argument, yet this is what a recent suggestion proposes to do as a palliative to the Chancellor's refusal to exempt hospitals from the payment of legacy duty. After calculating that the "considerable loss of revenue," feared by the Chancellor if exemption were granted, would amount only to £57,250 a year, the following plan is propounded:—"If a man leaves £10,000 to hospital A, the hospital only gets £9,000, and the Government gets £1,000; and the testator's estate is out of pocket £10,000. But if he will leave hospital A £9,000 free of duty, the hospital gets the same amount as before—viz., £9,000, and the Government only gets £900; and there still remains, out of the £10,000, £100 to be left, say, to the London Hospital, on which it will have to pay £10 duty. If my plan is followed the testator will have benefited two hospitals by £9,000 and £90 respectively, and the Government will have received only £910. His estate will be out of pocket £10,000," just as before. That is quite true, and every intending testator should be grateful for having it explained. But we do not believe that one testator in ten would take the trouble to think the matter out, or, if he did, to put the plan into practice. Institutional workers owe a very great debt to those benevolent impulses which have influenced so many testators on their behalf: every hospital possesses a cloud of witnesses to their force. But, as a whole, the testator is not influenced by intellectual appeals. The proof of this dogmatic assertion is to be found in the fact that so few testators leave their money to provide for new needs, while the vast majority leave it to support systems or foundations which are already on their legs, or in other words, which have proved their necessity, and therefore guaranteed their permanence, to the nation. If, however, events show us to have been false prophets as to the popularity of this plan no one will be better pleased than ourselves.

### A COURSE IN HOSPITAL ADMINISTRATION.

The Metropolitan Asylums Board announces a three months' course of lectures and demonstrations in hospital administration for medical men who are seeking to pass an examination for a Diploma in Public Health. Dr. E. W. Goodall will lecture at the Eastern Hospital, Homerton, N.E., on Tuesdays and Fridays at 2.30, beginning April 4; Dr. J. MacCombie at the North-Western Hospital, Hampstead, N.W., on Mondays and Thursdays at 5, beginning April 7; and Dr. J. E. Beggs, at the Grove Hospital, Tooting, S.W., on Wednesdays and Fridays at 5, beginning April 25. The fee for the course is three guineas. This idea is worthy of extension to others besides medical men. Hospital secretaries and clerks, matrons, and members of house committees might well be given similar opportunities by the provision of courses of lectures more especially designed to fulfil their requirements. The time, indeed, may some day come when no one can enter for a hospital secretaryship without having attended some such course and obtained a satisfactory report for attendance and diligence.

### A HISTORY OF HOSPITAL APPEALS.

No one yet, so far as we are aware, has attempted a history of hospital appeal-writing, and yet from a curious as well as an historical point of view such a history would furnish many ideas as well as some amusement to institutional workers, and, indeed, to the general public. Indeed, the department of hospital history, save for Burdett's "Hospitals and Asylums of the World," and for those individual volumes of which Mr. Morris', Mr. Burford Rawlings', and Mr. G. C. Peachey's, of St. George's, are invaluable types, has been rather a neglected study till the past year or two. As a minor contribution to the history of appeals, we note the last of Sir Squire Bancroft's readings of Dickens' "Christmas Carol," which, it is interesting to record, was given on behalf of the Middlesex Hospital. Sir Squire Bancroft's readings of the famous "Christmas Carol" certainly mark an epoch in the history of appeals, and the date of the last, certainly has historical value in this connection. Originally, no doubt, Sir Squire Bancroft was following in the footsteps of Dickens, whose attempts to get into close quarters with his public form such a curious page in literary history and such a sad one in Dickens' own life. It is curious, by the way, to note that the Middlesex Hospital, which has developed so largely the "charity show" in all its forms, should have reverted for once to a reading from Dickens, which is probably, thanks to Sir Squire Bancroft's generosity, one of the earliest forms of entertainments on behalf of charity. The whole subject invites us to stray into its many by-paths and later developments, but that temptation is the province of the future historian of hospital appeals, who we hope will make the best of this fascinating subject.

### DEATH OF A PANEL DOCTOR.

Mr. Thomas Chant, one of the first medical men to join the panel at Hackney, has died at his house in the borough. It does not appear in this case that death was due to overwork caused by the panel system. Mr. Chant, however, had only been some six months in Hackney, where he became known as an early supporter of the panel system. He qualified L.S.A. and M.R.C.S. in 1875, and his appointments included that of house apothecary at Bridgwater Infirmary. His medical education took place at the London Hospital.

### ANOTHER DEATH FROM "SICKNESS BENEFIT."

Is dying by starvation intended to be included among the sickness benefits afforded by the Insurance Act? One is compelled to ask this question in all its seriousness after reading how an insured man has died of starvation after receiving Poor-Law relief under the following circumstances. The death of an insured colliery worker, named Moore, who, with his wife, recently went from Mexborough to live at Chertsey, was reported to the Burton-on-Trent Guardians last week. Three weeks ago, it is reported, Moore fell ill with pleurisy and double pneumonia. After a fortnight had passed without him being able to obtain sickness benefit under the Insurance Act, the doctor gave him an order on the relieving officer, and endorsed it, "Dying by starvation." The relieving officer at once gave the man's wife 7s. 6d., but ten minutes after Mrs. Moore returned home her husband died. From the man's insurance card it appears that he was entitled to 27s. 4d. This case, in all its naked horror, appears to have aroused the local insurance superintendent to make an explanation. His defence, for that is what it amounts to, is this: The man, states the superintendent, failed to notify his change of address, which caused a delay in obtaining his card. When this was received the superintendent immediately notified the Commissioners in London on Monday last week. He received no reply. Without prejudging any "reply" which the Commissioners may see fit to make—and official replies, as question time in the House of Commons exists to prove, are exceedingly easy to manufacture—we think that the authors of the Insurance Act and the administrators of the "benefits" and regulations connected with it will find it not very easy to escape from the fact that an insured sick person, to whom 27s. 4d. was owing, has died of starvation, which even the relieving officer arrived too late to prevent. We record this as the latest fruit on the sinister tree of panel servitude and sickness "benefit."

### LISTER'S VIEWS ON HOSPITAL CONSTRUCTION.

In delivering the Foundation oration before University College Union Society on "Lister and his Work," Sir Rickman Godlee, P.R.C.S., said: "Lister never worked in a palatial hospital and equally good results were obtained by any one who had grasped his idea and carefully acted upon it in old and crowded wards or in the cottages of the poor." If these words come to the notice of any hospital architect, still more if they gain more than a passing glance from a modern hospital secretary or committee-man they may well give him pause. For they mean nothing less than a revolution in hospital construction and are a damaging criticism on much institutional building of the last twenty years. For what is their implication? It is simply this: that where the principle of antisepsis is properly carried out there is, in fact, no need for those expensive devices from the popular rounded corner to the impermeable wall or floor, which seek mechanically to guard against risks which aseptic surgery properly carried out makes non-existent. That, indeed, is why "equally good results" are obtained in old provincial or infirmary operation rooms as in the most extravagant glass-tiled theatre in London. It is not the theatre but the surgeon who secures a good result, and an argument can even be urged against extravagant construction as tending to blunt the operator's sense of responsibility by mechanical contrivances which can never under any circumstances take the place of Listerian cleanliness in the surgeon himself and in his assistants. Expensive and elaborate construction, with the host of fads and devices which they entail, is seen to be an anachronism in any hospital where the principle of antiseptic surgery is carried out. What a light this throws on the enormous expenditure on building of the last fifteen or twenty years! Were its truth grasped by hospital committees generally the building and designing of the next ten years would cost at least half what will probably be subscribed and spent upon it. This, the second great deduction from Lister's teaching, has so far fallen practically on deaf ears.

### THE BUSINESS SENSE OF A MENTAL PATIENT.

An interesting judgment by the President of the Probate Division of the High Court has pronounced for the validity of a will and codicil which was drawn up by a lady patient who had lived for several years in Bridlington House Mental Hospital, Bristol. Miss Catharine Maddock, the testatrix, who died in May 1911, in October 1880 wrote an admirably expressed business letter to the Commissioners in Lunacy, asking whether a testament or will would be "legal and binding made by me who have lived thro' 9 years in Bridlington Mental Hospital." She then explained to the Commissioners that she could not get a straightforward answer from her brother, to whom she had entrusted her papers when her illness began, nor from Dr. Fox, though such an answer was quite necessary now, for various adopted children were coming from India, in order "to secure to them the rightful possession of property long bequeathed to them." She received a reply to the effect that an inmate of a mental hospital in certain circumstances could execute a valid will, which, we are glad to think, in her case has now been finally settled. Few persons in such a difficult situation as Miss Maddock, even if outside a hospital, could have written a more admirably styled and terse business letter than that from which we have quoted. A perusal of even these extracts should give many laymen a new idea of what constitutes a mental patient.

### PROPOSED NEW HOSPITAL FOR DEVON.

Subject to the consent of the Tiverton authorities immediately concerned, and to that of the Local Government Board, the Devon County Council propose to build a consumption hospital with twenty beds contiguous to the present isolation hospital on the Halberton Road. The institution when completed will be under the management of the Joint Hospital Board, which will be paid by the county authority from 28s. to 30s. per week per bed, whether the beds are occupied or not. Mr. A. T. Gregory, the Mayor of Tiverton, explained that the hospital will be for the eastern part of the county, but that it was too early to state whether or not insured persons will or will not be admitted. While appreciating the importance of this question, which was at once asked by Mr. Nott, we are bound to point out the supreme importance of appointing as medical officer a medical man of special experience, not merely of tuberculosis, but also of the administration of consumptive patients. There is always a danger when a public authority—like, for instance, the Metropolitan Asylums Board—takes over, as in this case, the control of an institution of different scope from the majority of those previously under its control, that the new posts to be created will be regarded rather as rewards for previous service than as new offices demanding new and specially trained officials.

### THE KING'S FUND AND AN OLD-AGE PENSION.

A remarkable instance of the way in which King Edward's Hospital Fund for London has impressed itself on the general public at large as the best channel for charitable subscriptions from those who have learnt to live up to the Fund's motto of personal service in the days of health for the cause of the sick, is provided by the following. The Fund's honorary secretaries have received a letter from a subscriber, a retired governess now in receipt of an old-age pension, containing the following terse biography: "By the aid of my old-age pension I am able to subscribe my mite of 5s. to King Edward's Hospital Fund. As I used to say, do not trouble to remind me, I am only too glad to forward the money when I have saved it." It appears that the lady, who for many years subscribed to the Fund out of an income of 15s. a week, at the age of sixty-eight was obliged once more to start earning her living. Now, however, an old-age pension has enabled her to renew her annual subscription. We wonder how many other of the Fund's subscribers vie with this lady in giving what is virtually one week's income to the Fund.

### THE HOSPITAL NOVELIST.

Hospital men, as we have often lamented, have been slow to venture into the domain of authorship, though every institution, as the growing number of hospital histories is beginning to show, is packed with the stuff from which literature is made. Nevertheless, in spite of this, the novel of hospital life has yet to be written. There is no field so full of possibilities for combining all the arts of the born storyteller as institutional work, and yet the school is the only institution which has produced a classic in fiction. Now, however, that Mr. Morris, Mr. G. C. Peachey, and Mr. Burford Rawlings have shown the way, and that medical men have become known as capital descriptive writers of travel or globe-trotting, we may hope that hospital life will soon find its Hughes or its Dickens also. As such a hope is a dear one to editors, who have seen the material botched and turned to scurrility by the baser sort of sensation-mongers so often as ourselves, we make no apology for drawing the attention of hospital workers to the claims of the Royal Literary Fund. This is not an anti-climax; for though nobody wishes to see a medical, let alone a hospital, author in want of money, the best literature seems to be happier in the slum than in the square; and if we are hoping for the hospital novelist we must not shut our eyes to the fact that he may be a man whose literary gifts have damned him as an administrator to save him as the observer and recorder of the administrative life. If so the institutional world may well have cause to be grateful to the Royal

Literary Fund, which was established in 1790 to help authors and their families in distress. The Fund, then, may have prophetic as well as retrospective claims to our regard.

### A HOSPITAL RADIUM FUND.

A Hospital's recent advertisement for radium has been followed up by a rather original appeal from Sir David Salomons, who quotes five specific appeals from London hospitals for this substance. He suggests that the nation, which is now "assisting at great cost the sick and wounded of the Turkish War," might purchase some of the 500 milligrammes now "lying idle in a safe in London," or at least provide a munificent benefactor "to present 100 to 200 milligrammes" to each of the five hospitals mentioned. Sir David modestly adds that he has "a small interest in the Cornish mine producing radium," and that "a few friends, with myself, secured this source of production in order to save it for this country and not for profit. Should the latter arise it will probably come from the tin in the mine." If we remember aright, though he now enforces the appeals of London institutions, Sir David Salomons has presented some important gifts of radium to his local institution, the General Hospital, Tunbridge Wells.

### A LESSON FROM THE RADIUM INSTITUTE.

Hard upon this appeal comes the news that Sir David Salomons has himself given radium to the value of £500 to the Cancer Hospital, Brompton. The gift is made subject to certain reasonable conditions. The hospital may not allow the radium to be used on their premises for any except in-patients or out-patients of the hospital—that is to say, no private patients may be treated with this radium on the hospital premises. But "from time to time and without favouritism" they may at their discretion allow the radium to be lent to any doctor, provided that the radium is not to be borrowed for long periods, and that the hospital may refuse applications for such loans without assigning any reason. Whenever such loans are made, they are to be free if the doctor's patient is poor; but otherwise charges up to one pound may be levied, divisible equally between the doctor who borrows the radium and the hospital funds. This latter provision is, in our opinion, a mistake; the whole charge should go to the hospital, and the doctor should be left to make his own terms with the patient for his services.

### PRESENTATION TO A PROVINCIAL SECRETARY.

Major Kingsley O. Foster took occasion at the recent annual meeting of the Reigate and Redhill Hospital to present to Mr. T. Painter, the late secretary of the institution, a cheque from the governors for £65 11s. Major Foster remarked that Mr. Painter had been their secretary for some eighteen years, and that he had always been most useful to the board of management, and also that he had never failed to work hard for the hospital on a very small salary. Mr. Painter, in acknowledging the gift, remarked that he was sorry he could not relinquish the position with a good balance in hand, as when he took over the position there were £110 to the hospital's credit. He had worked under three different chairmen, and only twelve members of the present committee and five of the board of management were there when he commenced the secretaryship in 1895. At that time there were only four medical officers; now there were six, and four consulting medical officers. Mr. W. E. Bartlett is his successor in the secretaryship, and it is pleasant to record this appreciation of quiet steady work, which is an example of that heavy mass of routine responsibility which still often goes unacknowledged in days when the successful administration of hospitals has come to be regarded —such has been the success of the voluntary system—almost as a matter of course.

### VISITORS AT INFIRMARY DEATH-BEDS.

The medical officer to the workhouse of the Newtownards Board of Guardians, Dr. J. M. Warnock, has issued a reply to Lady Hermione Blackwood's criticism of the "exceptional severity" of its rules as regards the visiting of dying patients by their relatives. Of the two examples adduced by Lady Hermione Blackwood we will take the first, in which she stated that a child of six, after having been for three weeks with scarlet fever in the infirmary, died there without her mother being allowed one glimpse of her during that time. Her ladyship then referred to the rules in the Belfast Infirmary, which, she said, allowed parents to view infectious children through a glass, and to the "danger list" which is given to the infirmary porters in London, so that the relatives may be summoned at all hours. Dr. Warnock replied that the child's death was quite unexpected, she having shown previous signs of recovery; and that her mother was informed, but arrived just too late. It was one of those cases of sudden collapse that no one could have foreseen. But such cases illustrate the difficulty of allowing the friends of infectious persons relatively free access to them when they show signs of recovery if the public health is to be safeguarded. We think this fairly disposes of the criticisms to which we have alluded, though such criticisms do good service from time to time in explaining the practical difficulties attending the admission of visitors, and show how sometimes it is impossible to avoid a charge of inhumanity however complete may be its subsequent refutation.

### VERONAL AND THE POISONS SCHEDULE.

It is no surprise to learn of the addition to the Poisons Schedule of veronal and other hypnotic drugs without further delay. After consultations between a representative of the Council of the Pharmaceutical Society and Privy Council officials a way out of the difficulty has been found, and the Pharmaceutical Council has submitted a new form of wording for the approval of the Lords of the Council. The difficulty is due to the fact that diethyl-barbituric acid is sold under a variety of trade names such as veronal, proponal, and medinal, and what increases the difficulty is that other names may be invented for similar substances. Hitherto technical language has not been employed in the Schedule nor have trade names been mentioned, but developments of synthetic chemistry render it necessary to make an innovation. The form of words proposed therefore includes diethylbarbituric acid, and derivatives of barbituric acid under whatever names they may be known; tetronal and trional are also to be added to the Schedule. Till this decision the only synthetic hypnotic included was sulphonal.

### THE TECHNIQUE OF REPORT DRAFTING.

ONCE again admiration compels us to advise every committee, which desire to see what a model hospital report can be made to be, to secure the latest, just issued by the Leicester Royal Infirmary. The attractive presentation of facts and figures is an art in which only of late years have we succeeded in persuading the generality of institutions to seek proficiency. Having dealt on previous occasions with the main facts in the attractive handling of statistics which this report reveals, this year we will draw attention merely to two minor details. One is the map which the report contains showing from what places unlimited by county boundaries the institution's patients are drawn. This not only appeals to subscribers by illustrating the wide range from which the sick come to the hospital's doors; it also represents by a kind of mental landscape the vital part which the hospital plays in the life of the county area as a whole. It is thus shown to be the capital of a great sick province. The second point is merely this, that even the blank spaces on the receipt and expenditure pages are not wasted. Within four lines each vacant space bears a different inscription, such as this: "£7,400 is still required to complete the reconstruction of the children's hospital." This is a small thing, but indicates the meticulous care and thought which have slowly built up this institution's annual report into what is probably the best in the country.

### GIFTS TO THE HOMŒOPATHIC HOSPITAL.

THE London Homœopathic Hospital, Great Ormond Street, has received an anonymous cheque for £10,000—£9,000 towards the debt to the bankers (amounting to £16,675), and £1,000 for a bed for the use of gentlewomen of small means who are not eligible for the ordinary free beds. At a meeting at which this was announced Lord Donoughmore, the chairman, added that Lord Dysart had promised to give the last £1,000 if the whole sum of £16,675 due to the bankers was raised by December 31 next. Mrs. Washington Epps had expressed her intention of giving £750 for a cot in the children's ward in memory of the late Dr. Washington Epps, who was for thirty-eight years an active member of the visiting staff.

### THE FOUNDER'S GIFTS TO TROPICAL MEDICINE.

SIR ALFRED JONES, even in these days of millionaires' unloading, has created surprise by bequeathing to medical and educational charities not far short of a quarter of a million. On Monday the scheme for carrying out the objects of the will was sanctioned at the Chancery Court of Lancashire sitting at Liverpool. These objects include £40,000 to the Liverpool School of Tropical Medicine, and a further £40,000 when the annuities payable out of the estate cease. The former is to form a fund to be called the "Sir Alfred Lewis Jones Bequest," and is to be devoted to defraying the cost of a new wing or ward to the Liverpool Royal Infirmary for persons suffering from tropical diseases to be called after the testator; to the erection of new premises in Liverpool for the study of tropical medicine, also to be permanently associated with his name; to the erection and equipment of a laboratory in Sierra Leone to be called the "Sir Alfred Lewis Jones Tropical Laboratory"; while the residue of the gifts is to be used as a permanent endowment. The sum of £10,000 is left for a hospital at Garston and £5,000 to the Liverpool Central Relief and Charity Organization Society, to be available for the relief of poor relations of the testator. This is one of the rare cases in which death and life vie with one another to express the creative ideas of the same man.

### SEPARATE THEATRE FOR SEPTIC CASES?

EFFORTS are being made by the Camberwell Board of Guardians to induce the Local Government Board to sanction the erection of a small separate theatre for septic cases. Dr. Keats, the medical superintendent, pointed out the necessity for the separate theatre, mentioning that on one day seven operations were performed requiring septic treatment. The Local Government Board, however, have informed the Guardians that a separate operating room for septic cases is unnecessary, if proper precautions are taken. It was practically impossible, the Board added, to maintain a clear division between septic and non-septic cases. It did not appear that the number of operations in the infirmary could be so great as to justify on that ground alone the expense of providing a separate room. Dr. Keats, in reply, points out that the danger to patients would not be so great if the nursing staff at the Infirmary was similar to that in a general hospital, but unfortunately they had neither an adequate medical nor nursing staff.

### EASTER AND THE DRUG MARKET.

As is usually the case on the eve of the Easter Holidays business in drugs is quiet. The market for cod-liver oil, however, is being followed with interest; present quotations being almost three times as high as the lowest price touched last year; there seems to be a prospect of a further advance. As was anticipated the price of cocaine hydrochloride has been reduced by eightpence per ounce. Quotations for acetyl-salicylic acid are also lower. A further advance has taken place in the price of citric acid, also in seidlitz powder and tartarated soda. The position of opium is practically unchanged, and very little business is being done. Quicksilver has been reduced in price. Almond oil is slightly dearer.

